# Metastatic Heterogeneity of Breast Cancer: Companion and Theranostic Approach in Nuclear Medicine

**DOI:** 10.3390/cancers12040821

**Published:** 2020-03-29

**Authors:** Christopher Montemagno, Gilles Pagès

**Affiliations:** 1 Département de Biologie Médicale, Centre Scientifique de Monaco, 98000 Monaco, Monaco; gpages@unice.fr; 2 Institute for Research on Cancer and Aging of Nice, Centre Antoine Lacassagne, CNRS UMR 7284 and INSERM U1081, University Côte d’Azur, 06200 Nice, France

**Keywords:** breast cancer, metastasis, nuclear medicine, theranostics

## Abstract

Breast cancer is the most common malignancy in women throughout the world. Metastatic dissemination to vital organs is the leading cause of breast cancer-related deaths. The treatment of metastases is mainly based on the primary tumor characteristics. However, breast cancer metastases exhibit high heterogeneity leading to different prognosis and therapeutic responses. Getting access to phenotype of metastases would allow better management of patients. The advent of theranostics in nuclear medicine has opened new opportunities for the diagnosis and treatment of cancer patients. The aim of this review is to provide an overview of current knowledge and future directions in nuclear medicine for therapeutic management of metastatic breast cancer patients.

## 1. Introduction

Breast cancer is the most common female malignancy, accounting for more than 30% of all malignant tumors in women [1]. In 2018, 2 million women were diagnosed with breast cancer [2]. Breast cancer is a complex and highly heterogeneous disease consisting of various subtypes. It is classified into human epidermal growth receptor 2 (HER-2), luminal A, luminal B and basal-like, based on histological and molecular features [3,4]. The prognosis of breast cancer patients is highly dependent of the breast cancer genotype; as patients with HER2 or basal-like cancers have worse prognoses compared to patients with luminal A or luminal B breast cancers [5]. Breast cancer prognosis is also highly dependent of the disease stage at the time of diagnosis. The five-year relative survival of early-stage breast cancer is 99%, whereas the prognosis of patients with metastatic breast cancer is unfavorable with a five-year survival rate of 25% [6]. Moreover, among women initially diagnosed without metastasis, 20% to 30% will develop a metastatic disease during the next five years [7]. Metastatic breast cancers are treated by chemotherapy or targeted therapy according to primary tumor features. However, 20% to 45% of metastases exhibit different phenotypes as compared to the primary tumor [8]. Getting access to metastases phenotype would define a more accurate treatment, depending on the molecular characteristics of these lesions. Metastases are rarely accessible to biopsy. Hence, molecular imaging is of great interest. Nuclear medicine is the only molecular imaging technique available in clinical practices. In addition to accurate phenotype acquisitions, nuclear medicine allows promising therapeutic opportunities to treat a large panel of cancers including breast [9]. 

## 2. Metastatic Dissemination of Breast Cancer

### 2.1. Generalities

Metastasis is the general term used to describe the spread of cancer cells from the primary tumor to distant organs. The vast majority of cancer-related deaths arise from the metastatic spread which is responsible for 90% of cancer-related deaths [10]. The process of metastatic dissemination remains poorly understood. When the diagnosis of breast cancer is established, 5% of patients already present a metastatic disease. Despite therapeutic advances, 20% to 30% of patients will relapse towards a metastatic disease [7,11]. The ability of tumor cells to disseminate, which is closely linked to overall survival, is dependent on the tumor subtype. Patients with basal-like and HER2-positive tumors have the shortest metastasis-free survival and overall survival in comparison to patients with luminal A and luminal B subtypes [3,5,12]. 

### 2.2. Metastatic Dissemination Pattern of Breast Cancer 

Breast cancers preferentially spread to the following organs: liver, lung, bone and brain. Breast cancer subtypes predict the preference to sites of distant metastasis [13,14]. Luminal A and B tumors preferentially colonize bone tissue with a lower rate of brain metastasis. HER-2-positive- and basal-like tumors exhibit higher rate of metastasis to the brain, the liver, bones and lungs [15,16]. The risk of developing brain metastases for the basal-like and HER2-positive subtypes is four times greater than for the luminal A/B subtypes [17]. Moreover, basal-like and HER-2-positive tumors are associated with early relapse [18,19].

### 2.3. The Metastatic Dissemination as an Early Event of Tumor Progression

While it is widely believed that distant metastasis is a late event in tumor progression, several studies have suggested an earlier occurrence of metastatic spread. The first highlight of this phenomenon comes from the analysis of scattered cells isolated from the bone marrow of patients with non-metastatic breast cancer; the presence of isolated tumor cells in the marrow increases the risk of relapse [20,21,22]. Accumulating evidences have shown that dissemination of tumor cells in mouse models of breast cancers as well as in the human disease can occur in preinvasive stages of tumor progression [23]. Similar findings have recently been found in preclinical models of pancreatic cancer and melanoma [24,25]. The results acquired from patients with precancerous breast lesions or carcinomas in situ are in agreement with these findings [23,26,27].

### 2.4. Metastatic Dormancy 

Most tumor cells that have carried out extravasation are unable to proliferate in the new environment and remain as micro-metastases. Micro-metastases can remain dormant for months or even years in secondary tissue. These micro-metastases constitute a residual disease characterized by the persistence of tumor cells in the body, which cannot be detected by conventional diagnostic techniques [28,29]. These cells do not exhibit histological and molecular features of transformed cells and are in a state of dormancy. The ability to isolate dormant cells is a difficult task. Hence, the mechanisms leading to metastatic dormancy remain poorly understood. The transition to clinically identifiable macro-metastases seems to coincide with the expansion of rare clones with specific genetic profiles [20]. Three molecular mechanisms characterize tumor dormancy: cell quiescence, angiogenic and immunological dormancy [30]. These dormant cells exhibit limited proliferation, an absence of Ki-67 labeling and apoptotic markers, linked to a reduced activity of PI3K-Akt pathway [31,32,33]. Moreover, dormant micro-metastases can also acquire a specific antigenic profile allowing them to escape the immune surveillance [34]. The regulatory mechanisms of dormancy remain to be investigated.

The early metastatic dissemination of breast cancer and the ability of tumor cells to remain quiescent should be considered as keys elements of metastatic relapse. A better understanding of metastatic processes will improve therapeutic strategies towards metastatic cancer. 

## 3. Heterogeneity of Breast Cancer Metastases

The therapeutic strategy for metastatic breast cancer relies on several parameters. It depends on the histological characteristics of the primary tumor, previous treatments and their tolerance, the site of metastatic lesions, the time to relapse and on predictive factors of treatment response (such as hormone-receptor expression or HER2 status) [35,36]. The molecular characterization of tumors is a key element in therapeutic management, but several studies have shown a lack of stability of the hormonal receptors and HER2 between the primary tumor and the metastases [8,37,38,39]. This observation underlines the need to reconsider the molecular status of secondary lesions to improve the treatment. Indeed, the loss of a predictive factor (HER2, ER, PR) can lead to the prescription of an unnecessary and potentially toxic treatment. Conversely, gaining a biomarker could offer interesting therapeutic opportunities for patients who did not initially receive a treatment directed against this target. 

In 2012, Lindström et al. demonstrated the instability in the clinically used markers. One third of patients experienced altered expression of hormone receptor, and 15% of HER2 expression during tumor progression [8]. Several authors further confirmed the discrepancy between the phenotype of the primary tumor and metastasis [40,41,42]; 15% to 40% of patients present a discrepancy in ER expression in the primary tumor and the metastases [8,43]. An equivalent mismatch stands for HER2 expression in up to 40% of patients [43,44]. The discrepancies in expression of progesterone and estrogen receptors and HER2 between tumor and metastases are observed in 33%, 20% and 8% of patients respectively [37]. 

The mechanisms leading to differential expression of these receptors have not been elucidated. Intra-tumor heterogeneity, molecular changes during the biological evolution of the tumor, the microenvironment or the selection pressure induced by therapies are probably involved as described for HER2 and hormone-receptor positive tumors following chemotherapy [45,46,47,48,49,50,51]. 

The heterogeneity between primary breast tumor and liver metastasis has recently led to a change in the therapeutic decisions, resulting as in an improvement of patients survival [52].

Currently, the molecular profile of the primary tumor and of metastases does not impact the patient’s therapeutic management. It is essential to reassess this therapeutic strategy and to discover new therapeutic targets for the management of metastatic breast cancers.

The assessment of the metastatic phenotype is critical for an optimal management of metastatic breast cancer patients. In this context, nuclear imaging allows non-invasive investigations of metastases that are not accessible to a biopsy.

## 4. Nuclear Medicine: Companion Marker and Theranostic Approach

### 4.1. Companion Approach

**Figure 1:**
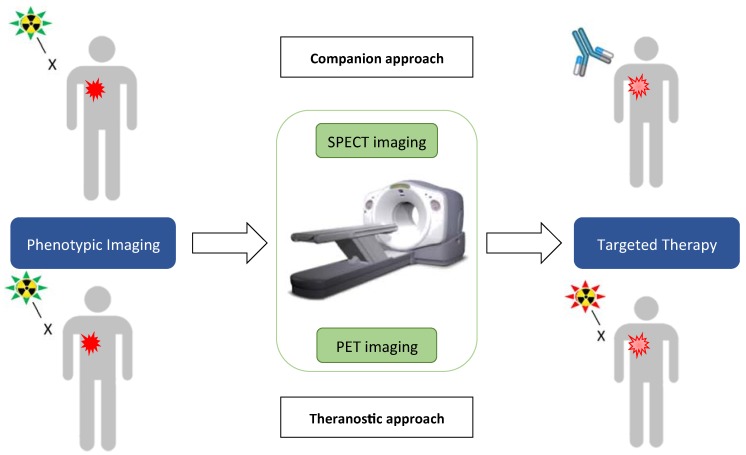
Companion and theranostic approaches in nuclear medicine. Phenotypic imaging (**left**) achieved using a PET- or SPECT-dedicated radiolabel compound can be followed by a non-radioactive therapy (**right**, **top**) or a radioactive therapy (**right**, **bottom**).

It is a diagnostic test selecting patients eligible for a specific treatment among those diagnosed for a specific disease according to their status for a predictive marker identified by the test (Figure 1) [53]. Currently, the prescription of targeted therapies is guided by the molecular characteristics of each patient’s tumor. For example, amplification of HER2 is systematically sought in patients with breast cancer, who may benefit from trastuzumab/Herceptin. In addition to the selection of patients eligible for targeted therapies, companion markers track the efficacy of these therapies. 

In the context of metastasis heterogeneity, nuclear imaging and the companion approach allows phenotyping of secondary lesions, in a non-invasive and very sensitive manner. Thus, developing companion tracers select patients who benefit from therapies targeting HER2 or hormone receptors, or other treatments directed against promising targets currently in development. Metastatic heterogeneity has been tested by imaging agents targeting HER2 [54,55]. A discrepancy between the primary tumor and the metastases was demonstrated in a non-invasive manner. Ulaner et al., have imaged primary HER2-negative tumors and HER2-positive secondary lesions with ^89^Zr-trastuzumab. Hence, anti-HER2 led to significant tumor regression in these patients [55].

More than 150,000 patients live with metastatic breast cancer in the United States [56]. If 80% of primary tumors are HER2 negative, and if a discrepancy is found in 8% of cases, 9600 patients do not benefit from HER2-directed therapy. Thus, HER2-directed agents could be a part of the therapeutic arsenal in a non-negligible number of patients. 

### 4.2. Therapies and the Era of Theranostics

Nuclear medicine images a target but also offers interesting therapeutic perspectives. Internal vectorized radiotherapy (IVR) is an intravenous radiotherapy using radiopharmaceuticals with a tumor tropism. It consists of specifically irradiating tumor targets using these molecules labeled by radionuclides β-emitting, α radiation or even Auger electrons, injected intravenously. The efficacy and toxicity of IVR depend on several parameters, including the nature of the vector, the target and the radioelement used. The radio-induced lesions by these radioelements target proteins, carbohydrates and lipids and nucleic acids (RNA and DNA). Several types of DNA damages can occur: single or double-strand breaks, DNA–DNA or DNA–protein bonds [57,58,59]. Radiation can also induce tumor apoptosis by activating cell death receptors [60]. The β-emitters are particularly used for targeted radiotherapy [61]. Their use in IVR therefore makes it possible to envisage the destruction of cells adjacent to the targeted cells. This “cross fire” mechanism and bystander effect can increase the effectiveness of the treatment [62]. The use of an alpha emitter in preclinical and clinical trials led to remarkable therapeutic efficacy in metastatic prostate cancer patients [63,64,65].

One promising and growing approach to treat cancer in nuclear medicine is theranostics, a combination of the diagnostic potential of nuclear imaging and the radiation therapy (Figure 1) [9]. Theranostics consists of using imaging to map cancer cells and then treat them in a targeted manner. The detection of potential targets predicts the relevance of a particular treatment. A typical example in theranostics is the use of β^+^ emitter 68-Gallium 68 (^68^Ga) tracers for diagnosis, followed by targeted therapy by labeling a similar or the same compound with β^−^ radionuclides, such as 177-Lutetium (^177^Lu) or 90-Yttrium (^90^Y). These three radioelements with similar chemical conformations can be complexed to the same group for the radio-labeling of the molecule of interest [66,67,68]. The great advances in the field of theranostics has led to remarkable progress in the management of patients with prostate cancer with the use of ^68^Ga-PSMA-617/^177^Lu-PSMA-617, or with neuroendocrine tumors with the use of ^68^Ga/^177^Lu-DOTA-TATE, while showing limited side effects [69,70,71]. Current clinical research shows a plethora of evidence towards the higher efficacy of alpha emitters [72]. Alpha emitter-labeled compounds have notably demonstrated remarkable therapeutic efficacy in metastatic prostate cancer [63]. 

This concept of theranostic medicine is perfectly suitable for management of metastatic breast cancer considering the tumor heterogeneity. By monitoring therapy according to the phenotype of these lesions, theranostic medicine can lead to more effective care while reducing unnecessary treatments.

## 5. Validated and Future Imaging Agents for Metastatic Breast Cancer

The assessment of differences between the primary tumor and the metastatic lesion, but also with different metastases in the same or different organs, requires several nuclear imaging tools with the aim of adapting treatment according to the expression of specific markers.

### 5.1. HER-2 Targeting Imaging Agents

Several clinical studies have been conducted with trastuzumab, labeled with ^64^Cu or ^89^Zr, in patients with HER2-positive metastatic breast cancer [73,74]. A nanobody targeting HER2 has been labeled with ^99m^Tc and ^68^Ga for SPECT and PET phenotypic imaging and with ^177^Lu for radiotherapy. Its theranostic potential has been extensively validated pre-clinically in mouse models of HER2-expressing breast tumors [75,76,77]. Its clinical transfer is in progress. Phase I in patients with HER2-positive breast cancer demonstrated the safety of the ^68^Ga labeled agent and its ability to target HER2-expressing breast tumors as early as 1 h after injection [78]. A phase II trial is underway in patients with HER2-positive metastatic breast cancer to evaluate the binding of this tracer in brain metastases (NCT03331601). HER2-positive lesions have been detected with the use of small molecules (affibody, ^68^Ga-ABY-025) [54].

### 5.2. Estrogen-Receptor Targeting Imaging Agents

^18^F-fluoro-estradiol or FES is a radiopharmaceutical agent targeting the estradiol receptor developed for PET imaging. The performance of FES-PET has been evaluated in several single-center studies between 1988 and 2011 [79,80]. The sensitivity of FES for bone and lung lesions is greater than 92% [81]. The performance of PET-FES has also been evaluated to predict the response to hormonal treatment in metastatic breast cancer; a predictive value of 79% and a negative predictive value of 88% were demonstrated [82]. The diagnostic accuracy and safety of ^18^F-FES was recently assessed successfully in metastatic breast cancer patients [83]. The staging assessment of patients with metastatic breast cancer should include ^18^F-FES-PET–CT, which is now available in clinical practice. 

### 5.3. Other Imaging Agents Currently in Development 

The discovery of the mechanisms and of the molecular actors in tumor progression has opened new opportunities for the management of several aggressive cancers, including metastatic breast cancer. Several targets have emerged and imaging agents targeting them are currently in preclinical or clinical development. Besides imaging agents directed against HER2 and estrogen receptors, some companion markers as well as theranostic agents could be promising for management of metastatic breast cancers (Table 1). 

Two promising theranostic couples represent relevant candidates for the therapeutic management of breast cancer patients: the gastrin release peptide receptor GRPR-targeting and PSMA-targeting agents. The expression of the GRPR has been reported in several cancers, including 72% to 96% of breast cancers [92,93]. Several imaging agents targeting this protein have recently been evaluated. The most promising NeoBOMB1, has been labeled with ^68^Ga and ^177^Lu and successfully evaluated in mouse models of prostate cancer [68]. A multicenter phase I study is ongoing with ^68^Ga-NeoBOMB1 in patients with gastrointestinal stromal tumors resistant to conventional therapies (NCT02931929). Theranostic agents targeting PSMA and, notably, ^68^Ga-PSMA-617 represent new tools of the therapeutic arsenal against metastatic breast cancer, since PSMA-based PET/CT imaging, which is now available in clinical practice is the procedure of choice in case of recurrence of prostate cancer [9,85,86].

Beside the theranostic approach, companion markers include PARP, mesothelin and PD-1/PD-L1-targeting imaging agents. 

On the basis of promising preclinical results, selective PARP inhibitors have been developed [94]. Among the imaging agents, PARP imaging with ^18^F-fluorthanatrace was successful in the breast cancer model [95]. This agent is currently under clinical investigation in several clinical trials on ovarian, prostate, pancreatic and recurrent or metastatic breast cancer (NCT03604315, NCT03334500, NCT03492164, NCT03846167). 

A third of breast cancers overexpress mesothelin, a 40-kDa membrane-associated glycoprotein playing a major role during tumor progression. Mesothelin is involved in the epithelium–mesenchymal transition, resistance to apoptosis and tumor invasion [96,97]. It is overexpressed in the most aggressive cancers and its expression is very low in physiological conditions. Hence, several molecules are currently in clinical phases to evaluate the therapeutic performance of its targeting in tumors expressing mesothelin [96]. Among companion markers, some monoclonal antibodies and nanobodies have been evaluated in clinical and in preclinical models of breast cancers [89,90,91]. Once available in clinical practice, such agents will allow selecting patients eligible to all these therapies. 

Finally, molecular imaging of the immune checkpoint receptor PD-1 and its ligand PD-L1 is investigated to monitor immune therapy targeting this axis. Some clinical trials with monoclonal antibodies and nanobodies are in clinical phases for the non-invasive imaging of PD-1/PD-L1 [87]. The chemokine receptor is also frequently overexpressed in invasive breast cancer and the diagnostic performance of CXCR4-directed PET imaging have been successfully assessed in recurrent breast cancer [88]. 

Other targets such as the epidermal growth factor receptor (EGFR) or Mucin-1 represent relevant targets for this theranostic approach [98,99,100]. 

## 6. Conclusions

The heterogeneity between the primary tumor and a secondary lesion raises the problem of the management of metastatic breast cancer based on the molecular markers of the primary site. Therefore, the phenotype of each lesion must be determined to personalize the management of each patient. The era of theranostic in nuclear medicine and its considerable contribution to precision is a key methodology of cancer treatment in the next decade. 

## Figures and Tables

**Table 1:** Targets and radioligands for phenotypic imaging of metastatic breast cancers.

Molecular Target	Expression in Breast Cancer (%)	Theranostic Molecule	Clinical Trial	References
**PARP**	45%	^18^F-fluorthanatrace	Yes	[84]
**Gastrin-Releasing**	75.8%	^68^Ga-NeoBOMB1	Yes	[68]
**Peptide Receptor**		^177^Lu-NeoBOMB1	Yes	
**PSMA**	60%	^68^Ga-PSMA-617	Yes	[71,85,86]
		^177^Lu-PSMA-617	Yes	
**PD-1/PD-L1**	29% to 50%	^89^Zr-Pembrolizumab	Yes (Phase II)	[87]
		^89^Zr-Atezolizumab	Yes (Phase I)	
		^18^F or ^89^Zr-Adnectin	Yes (Feasibility)	
		^99m^Tc-Nb	Yes (Phase I)	
**CXCR-4**	56%	^68^Ga-Pentixafor	Yes	[88]
**Mesothelin**	30%	^89^Zr-mAb (MM0T0530A)	Phase I	[89,90,91]
		^111^In-Amatuximab	Phase I	
		^99m^Tc-A1	No	
